# Caregiver Preferences for AI-Supported Telemedicine in Pediatric Palliative Care: A Discrete Choice Experiment

**DOI:** 10.1177/08258597261458091

**Published:** 2026-06-10

**Authors:** Carlos Antonio Godoy Junior, Lucia Peñarrubia-San-Florencio, Silvia Ricart, Sergi Navarro Vilarrubí, Maria Rimblas Roure, Cristina Ruiz-Herguido, Arnau Valls-Esteve, Luis Pilli, Carin Uyl-de Groot, William Ken Redekop, Welmoed Kirsten van Deen

**Affiliations:** 1 Erasmus School of Health Policy and Management, 6984 Erasmus University Rotterdam, Rotterdam, The Netherlands; 2 Pediatric Palliative Care and Complex Chronic Patient Service, 16512 Sant Joan de Déu Hospital, Esplugues de Llobregat, Barcelona, Spain; 3 Research Institute Sant Joan de Déu, Esplugues de Llobregat, Barcelona, Spain; 4 Faculty of Nursing and Physiotherapy and GESEC, Universitat de Lleida, Lleida, Spain; 5 Sant Joan de Déu Foundation, Esplugues de Llobregat, Barcelona, Spain; 6 Innovation in Health Technologies, Research Institute Sant Joan de Déu, Esplugues de Llobregat, Barcelona, Spain

**Keywords:** palliative care, pediatrics, caregivers, telemedicine, artificial intelligence, patient preference, home care services

## Abstract

**Background:**

Asynchronous telemedicine may support home-based pediatric palliative care (PPC) by improving access to professional guidance and reducing caregiver uncertainty. Artificial intelligence (AI) may further enhance such services by supporting triage and workflow efficiency, yet evidence on how families prioritize specific features of AI-supported telemedicine remains limited.

**Aim:**

To quantify primary caregivers’ (PCGs) preferences for key characteristics of an asynchronous, AI-supported telemedicine program for children receiving home-based PPC.

**Methods:**

A discrete choice experiment (DCE) was conducted among PCGs of children enrolled in a tertiary PPC program. Participants completed 12 choice tasks involving trade-offs between telemedicine attributes: type of service (doctor- and nurse-supported telemedicine vs AI-supported telemedicine), response time to receive a reply from a clinician or nurse (6-48 h), and reduction in hospital visits. Preferences were analyzed using a mixed multinomial logit model.

**Results:**

Thirty-one PCGs completed the survey. Participation in a telemedicine service was preferred to the opt-out alternative. Response time to clinician or nurse feedback was the dominant driver of preferences, accounting for approximately 60% of decision-making. PCGs showed a modest but statistically significant preference for doctor- and nurse-supported telemedicine. Reduction in hospital visits was not a consistent driver of choices. Predicted uptake was high but declined markedly with longer response times.

**Conclusion:**

PCGs value telemedicine in PPC primarily for timely access to trusted clinicians rather than efficiency alone. AI-supported telemedicine appears more acceptable when it augments clinicians and shortens the time to human response. Responsiveness and continuity of clinician relationships should inform the design of future digital services in PPC.

## Introduction

Pediatric palliative care (PPC) aims to ensure the highest possible quality of life for children with life-limiting (LL) and life-threatening (LT) conditions and their families.^1^ While PPC can be delivered across multiple settings, many families prefer care at home, where the child can remain in a familiar environment and family life can be preserved.^2^ Home-based PPC, however, requires continuous coordination, timely clinical guidance, and psychosocial support, often under conditions of uncertainty and emotional strain.^2^

Sustaining home-based PPC is increasingly challenging. Specialized PPC professionals are scarce, care pathways are resource-intensive, and a growing population of children living longer with LL or LT conditions places additional pressure on services.^3,4^ These pressures have stimulated interest in digital innovations that could extend the reach of PPC teams without compromising care quality.

Telemedicine has emerged as a promising approach to support PPC delivery at home by facilitating communication between families and care teams, enabling remote symptom monitoring, and improving care coordination.^5,6^ More recently, artificial intelligence (AI) has been proposed to enhance telemedicine. Asynchronous, AI-supported telemedicine programs could allow families to submit questions or symptom updates at any time, while AI systems assist healthcare professionals with triage and clinical decision support.^7^

At the same time, introducing AI into PPC raises ethical, relational, and organizational concerns, including depersonalization of care, transparency, accountability, data privacy, trust, and the balance between automation and human judgment.^6,8,9^ These concerns may be particularly important in PPC, where care is emotionally sensitive and grounded in trust between families and clinicians.^10,11^

Although research on AI acceptability in healthcare is growing, evidence on family preferences in PPC remains limited.^8,12–14^ It remains unclear which features of AI-supported telemedicine programs families value most, which trade-offs they are willing to accept, and where resistance may arise. This study therefore aimed to elicit and quantify primary caregivers’ (PCGs) preferences for key characteristics of an asynchronous, AI-supported telemedicine program in PPC using a discrete choice experiment (DCE).

## Methods

### Study Design and Setting

A DCE was conducted to explore how PCGs of children receiving PPC value features of an asynchronous, AI-supported telemedicine program. DCEs are a well-established survey method and are more informative than ranking methods when the aim is to assess which features participants prefer and the trade-offs they are willing to make between service characteristics in hypothetical scenarios.^15–18^

The study focused on a potential home-based asynchronous telemedicine service through which families could submit questions or symptom updates via a digital platform and receive later responses from the care team.^19,20^ AI was conceptualized as supporting triage and common or nonurgent concerns while maintaining clinician oversight. The study was conducted at a tertiary pediatric hospital with an established home-based PPC program. At the time of the study, the service was under investigation as a proof-of-concept and had not yet been implemented in routine practice (Technology Readiness Levels 3-4).

### DCE Methodology

The DCE design followed best practice guidelines from the International Society for Pharmacoeconomics and Outcomes Research for conjoint analysis in healthcare,^21^ and reporting was informed by the DIRECT checklist for health DCEs (Supplemental File S1).^22^ Attribute development was informed by a targeted literature review on telemedicine and digital health in PPC,^23,24^ together with findings from our preceding qualitative study exploring caregiver perspectives on hybrid care models.^8^ Review of the candidate attributes involved a multidisciplinary team including PPC clinicians, nurses, digital health experts, and researchers with qualitative expertise (CG, WD). A summary of the candidate attributes considered during development, and the rationale for retaining the final attributes, is provided in Supplemental File S2.

Across the literature and qualitative work, key themes included timely access to professional advice, reassurance outside scheduled visits, preservation of professional involvement, and the potential to manage some concerns at home without hospital attendance.^8,23,24^ Based on their relevance to the study objective, salience to caregivers, conceptual distinctness, and feasibility for inclusion in a manageable DCE, 3 attributes were selected for the final experiment: (1) type of telemedicine service, (2) waiting time for a response from the palliative care team, and (3) reduction in hospital visits. Each attribute was assigned 2 to 4 levels reflecting current practice and plausible future telemedicine scenarios (Table 1).

**Table 1.** Attributes and Levels Used in the DCE.

**Attribute**	**Levels**
**Type of telemedicine service**	Telemedicine service with support from AI, doctor, and nurse Telemedicine service with support from doctor and nurse only
**Waiting time for healthcare provider response**	Within 6 h Within 12 h Within 24 h Within 48 h
**Reduction in hospital visits**	Avoids 2 of 10 visits Avoids 3 of 10 visits Avoids 4 of 10 visits Avoids 5 of 10 visits

Abbreviations: AI, artificial intelligence.

To support consistent interpretation, respondents received standardized descriptions of the telemedicine service scenarios. The AI-assisted telemedicine scenario was presented as a digital service allowing caregivers to submit questions or symptom updates and receive immediate automated feedback for common or nonurgent concerns. The AI was explicitly described as providing initial guidance only, without making diagnoses or treatment decisions, with all cases requiring further assessment escalated to a doctor or nurse, who retained full clinical responsibility. The doctor- and nurse-supported scenario was described as an online service without AI assistance or immediate automated feedback.

The number of attributes and levels was limited to reflect the expected small sample size typical of PPC research and to minimize cognitive burden.^25^ The experimental design estimated main effects only; interaction effects were not targeted. Choice tasks were generated in Ngene (version 1.4; ChoiceMetrics, Sydney, Australia) using a D-efficient design with small, fixed priors based on anticipated effect directions (Supplemental File S3).^26,27^

**Figure 1.**
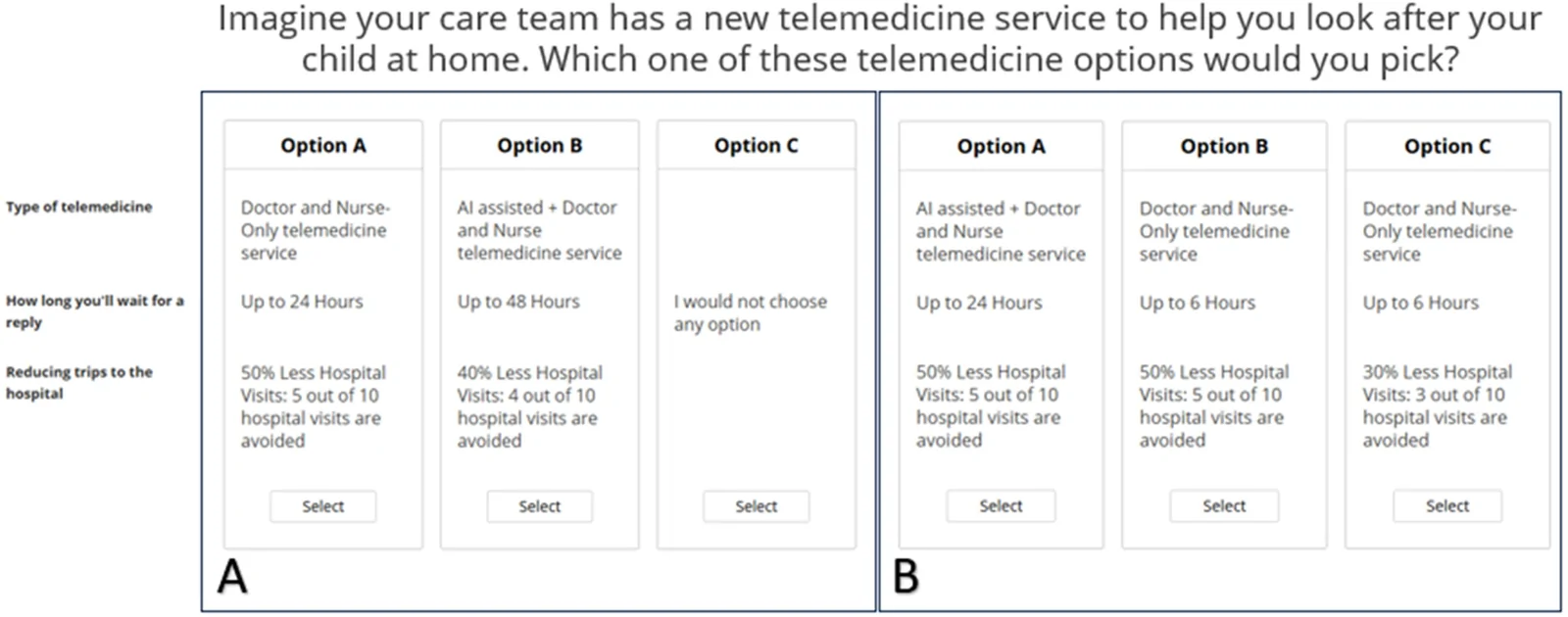
Examples of DCE task formats: (A) with an opt-out option and (B) without an opt-out option.

Abbreviation: DCE, discrete choice experiment.

The final design comprised 24 choice tasks, blocked into 2 versions so that each participant completed 12 tasks. Participants were assigned automatically to 1 of the 2 questionnaire blocks by the survey software, and choice tasks were presented in a fixed order within each block. The design was balanced across attributes and levels. To increase analyzable observations, a mixed task format was used: 6 tasks compared 2 telemedicine profiles plus an opt-out option, and 6 tasks compared 3 telemedicine profiles without an opt-out. Examples are shown in Figure 1.

### Survey Instrument

The survey was administered in Sawtooth Lighthouse Studio (version 9.15.4, Sawtooth Software, Provo, UT, USA). Beyond the choice tasks, the survey collected demographic and clinical information on PCGs and children, including caregiver characteristics, caregiving context, digital device use, confidence with online health tasks, child age, and care complexity indicators such as medical device use and emergency department visits. An English version is provided as Supplemental File S4.

Pretesting was conducted with 3 PCGs using a think-aloud protocol, a cognitive pretesting method in which participants verbalize their thoughts while completing the survey to identify issues with interpretation, wording, and task understanding.^28^ Based on feedback, minor revisions were made to simplify wording and clarify the instructions and presentation of the choice tasks. The experimental design was not changed. These participants were excluded from the final analysis.

### Participants and Recruitment

The target population consisted of PCGs of children enrolled in the hospital PPC program. The minimum sample size was estimated using the Johnson and Orme rule of thumb, N>500c/(t×a) , where *c* is the largest number of levels for any attribute, *t* is the number of choice tasks completed by each respondent, and *a* is the number of alternatives per task. In this study, c=4 , t=12 , and a=3 , resulting in N≥500×4/(12×3)=55.6 , which was rounded up to 56 participants.^29^ Recruitment occurred between September 2024 and May 2025 through in-clinic invitations, home visits, and email invitations via a secure institutional patient communication system. Participants received a secure survey link, provided electronic informed consent, and completed the survey without compensation.

The study received ethical approval from the ethics committee and adhered to the Declaration of Helsinki and applicable data protection regulations and General Data Protection Regulation (GDPR).^30^

### Statistical Analysis

All analyses were conducted using R statistical software (version 4.0.2; R Foundation for Statistical Computing, Vienna, Austria) and the Apollo package for discrete choice modelling.^31^ Preferences were first estimated using a multinomial logit (MNL) model.^32^ Attribute levels were modeled as categorical and dummy-coded,^33^ and the opt-out alternative was included as a separate alternative-specific constant to capture respondents’ preference for participating in the telemedicine program versus opting out.^34^ Model estimates (beta coefficients) indicate the direction and strength of preference for each attribute level, with statistical significance assessed using confidence intervals and *P*-values.

We subsequently estimated a mixed MNL (MMNL) model.^35^ All nonreference attribute levels were specified as random coefficients with normally distributed preferences to evaluate whether allowing for unobserved heterogeneity improved model fit. Random-coefficient models were estimated using simulated maximum likelihood with 500 Halton interindividual draws.

### Conditional relative importance, uptake prediction, and robustness analyses

Conditional relative importance (CRI) was calculated as the utility difference between the most and least preferred levels of each attribute and normalized to sum to 100%, representing each attribute's relative contribution to decision-making.^32^ Uncertainty was quantified using simulation-based uncertainty intervals derived from repeated CRI calculations across simulated parameter draws.

Uptake predictions were generated by simulating choice probabilities for selected telemedicine profiles using the estimated preference parameters.^35^ Scenario-based comparisons were used to interpret trade-offs between attribute levels. Marginal rates of substitution were not estimated because the DCE did not include a monetary or other suitable continuous trade-off attribute for that purpose.

Internal validity and robustness were assessed through post-hoc response-quality checks, including completion time and within-respondent choice variation. Sensitivity analyses reestimated models after excluding flagged respondents.

## Results

Of the 113 PCGs invited, 31 completed the DCE survey in full, with no missing or incomplete responses, yielding a response rate of 27.4%. Recruitment occurred over 8 months and included multiple reminder emails as well as active invitations during routine clinical consultations. Despite these efforts, additional recruitment proved infeasible, and data collection was therefore concluded with the available sample. All 31 complete responses were retained in the primary analysis; response-quality exclusions were explored in sensitivity analyses.

Participant characteristics are summarized in Table 2. Most respondents were mothers (81%), married or partnered (97%), and highly educated (64% held a university degree). Digital literacy was high, with 90% reporting daily use of digital technology and 81% expressing high confidence in completing online health forms. Most participants were born in Europe, lived within 20 km of the hospital, and shared caregiving responsibilities. The children receiving PPC spanned a broad age range.

**Table 2.** Sociodemographic and Caregiving Characteristics of Participating Respondents.

**Characteristic**	**Individuals, No. (%) (N = 31)**
**Primary caregivers**	
Relationship with the child	
Mother	25 (80.6)
Father	6 (19.4)
Age group (years)	
25-34	3 (9.7)
35-44	11 (35.5)
45 or older	17 (54.8)
Marital status	
Single	1 (3.2)
Married/partnered	30 (96.8)
Gender	
Female	25 (80.6)
Male	5 (16.2)
Other	1 (3.2)
Education level	
< High school	3 (9.7)
High school	3 (9.7)
Technical/professional	5 (16.1)
University	20 (64.5)
Frequency of using digital devices	
Never	1 (3.2)
Occasionally	2 (6.5)
A few times per month	0 (0.0)
A few times per week	0 (0.0)
Every day	28 (90.3)
Confidence in completing medical forms online	
Not at all	0 (0.0)
A little	3 (9.7)
Somewhat	3 (9.7)
Quite a bit	15 (48.4)
Extremely	10 (32.3)
Employment status	
Employed full-time	7 (22.6)
Employed part-time	6 (19.4)
Compassionate leave	4 (12.9)
Unemployed	9 (29.0)
Retired	0 (0.0)
Other	5 (16.1)
Region of birth	
Europe	22 (71.0)
America	6 (19.4)
Asia	3 (9.7)
Distance from home to hospital	
< 20 km	18 (58.1)
20-50 km	8 (25.8)
51-100 km	3 (9.7)
> 100 km	2 (6.5)
People involved in caregiving	
Only respondent	1 (3.2)
1 other person	21 (67.7)
2 or more	9 (29.1)
Other children in household	
0	12 (38.7)
1	14 (45.2)
2 or more	5 (16.1)
**Children**	
Age group (years)	
0-1 year	5 (16.1)
2-4 years	3 (9.7)
5-10 years	7 (22.6)
11-15 years	9 (29.0)
16+ years	7 (22.6)

Preference estimates were first explored using an MNL model and then reestimated using an MMNL model to account for unobserved preference heterogeneity. Coefficient directions were similar across models, but the MMNL provided a better fit and was therefore retained for interpretation (Table 3).

**Table 3.** Preference Estimates from the Multinomial Logit and Mixed Multinomial Logit Models.

**Attribute (Base Level)**	**Level**	**MNL β (Robust SE), [95% CI]**	**MMNL Mean β (Robust SE), [95% CI]**
**Main effects**			
**ASC**	Opt-out	−3.20 (0.49)***, [−4.16 to −2.25]	−3.83 (0.61)***, [−5.03 to −2.63]
**Type of telemedicine service** (AI, doctor, and nurse)	Doctor and nurse only	0.41 (0.19)**, [0.04 to 0.79]	0.92 (0.37)**, [0.18 to 1.65]
**Waiting time** (Within 6 h)	Within 12 h	−0.58 (0.20)**, [−0.96 to −0.19]	−0.62 (0.32)*, [−1.25 to 0.02]
	Within 24 h	−0.94 (0.32)**, [−1.57 to −0.31]	−1.37 (0.47)***, [−2.28 to −0.46]
	Within 48 h	−1.60 (0.44)***, [−2.47 to −0.73]	−3.55 (1.15)***, [−5.81 to −1.30]
**Reduction in hospital** visits (Avoids 2 of 10 visits)	Avoids 3 of 10 visits	−0.45 (0.32), [−1.07 to 0.18]	−0.45 (0.34), [−1.12 to 0.22]
	Avoids 4 of 10 visits	−0.78 (0.41)*, [−1.58 to 0.03]	−1.22 (0.44)***, [−2.09 to −0.35]
	Avoids 5 of 10 visits	−0.60 (0.46), [−1.50 to 0.30]	−0.55 (0.63), [−1.79 to 0.69]
**Standard deviations (MMNL only)**			
**Type of telemedicine service**	Doctor and nurse only	—	1.49 (0.45)***, [0.61 to 2.36]
**Waiting time**	Within 12 h	—	0.04 (0.23), [−0.42 to 0.50]
	Within 24 h	—	1.28 (0.37)***, [0.56 to 2.00]
	Within 48 h	—	2.87 (0.93)**, [1.06 to 4.69]
**Reduction in hospital visits**	Avoids 3 of 10 visits	—	−0.16 (0.35), [−0.84 to 0.52]
	Avoids 4 of 10 visits	—	1.16 (0.46)**, [0.27 to 2.06]
	Avoids 5 of 10 visits	—	4.51 (1.73)**, [1.13 to 7.89]
**Model fit**			
**LL (final)**		−292.64	−252.50
**McFadden pseudo-R^2^**		0.28	0.38
**AIC**		601.27	535.01
**BIC**		632.62	593.79

Abbreviations: MNL, multinomial logit; MMNL, mixed multinomial logit; SE, standard error; CI, confidence interval; LL, log-likelihood; AIC, Akaike information criterion; BIC, Bayesian information criterion.

*P < .10, **P < .05, ***P < .01.

In the MMNL, respondents generally preferred participation in the telemedicine program over opting out, as reflected by the negative opt-out coefficient. Compared with AI-supported telemedicine, a doctor- and nurse-supported service was preferred (β = 0.92; *P* = .01). Waiting time emerged as the most influential attribute. Relative to a response within 6 h, utility decreased for a response within 24 h (β = −1.37; *P* < .01) and within 48 h (β = −3.55; *P* < .01), whereas the estimate for 12 h was borderline significant (β = −0.62; *P* = .06).

For reduction in hospital visits, only the 40% level differed significantly from the reference level. Overall, the coefficients for reduction in hospital visits were negative, suggesting that larger reductions in hospital visits were not positively valued on average within the range presented. Significant standard deviations for several random parameters, including telemedicine type (SD = 1.49; *P* < .01), waiting time within 24 h (SD = 1.28; *P* < .01), waiting time within 48 h (SD = 2.87; *P* < .01), and reduction in hospital visits by 40% (SD = 1.16; *P* = .01) and 50% (SD = 4.51; *P* < .01), indicated meaningful heterogeneity in preferences across respondents.

**Figure 2.**
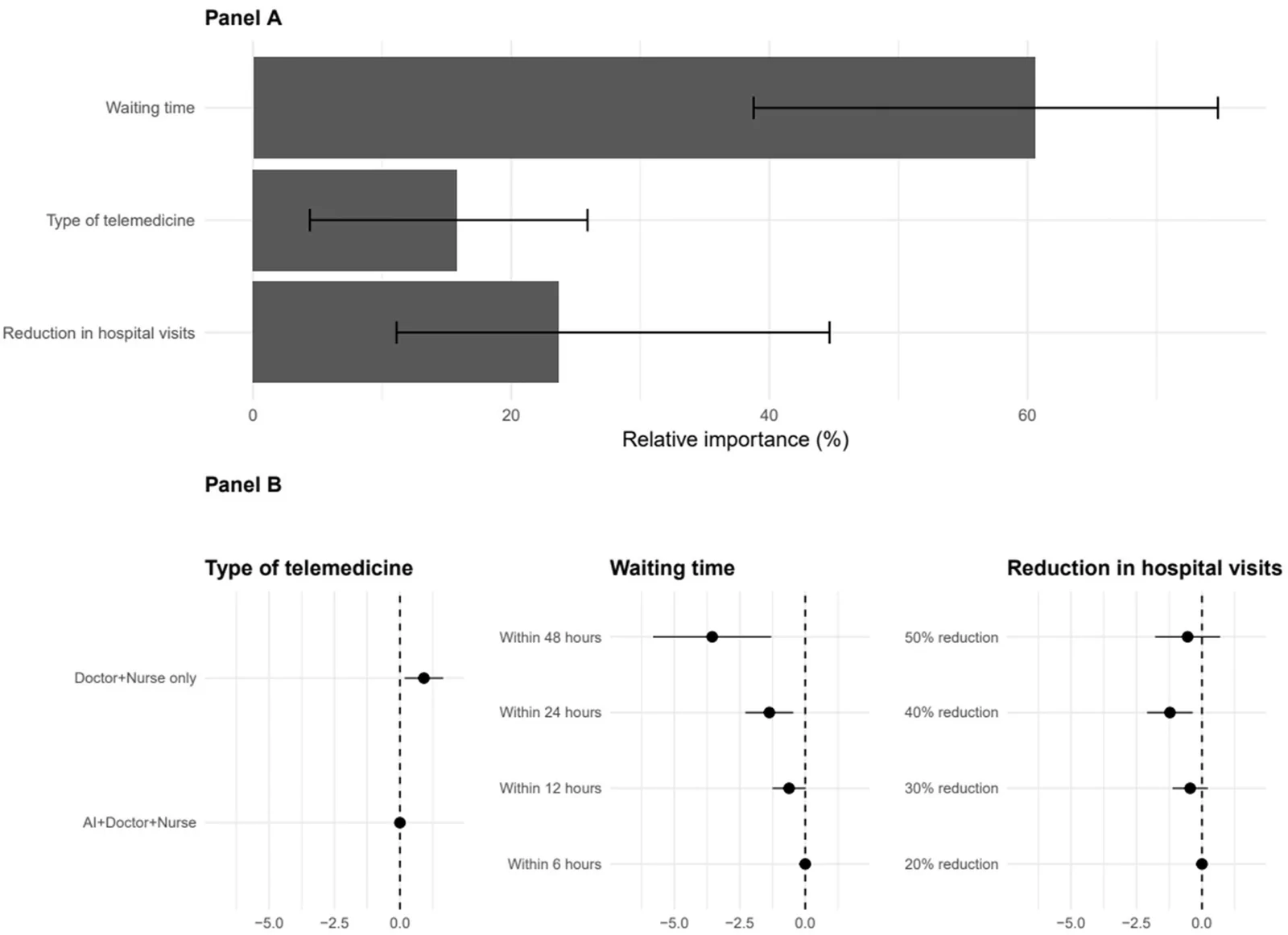
Conditional relative importance (CRI) and comparative attribute-level effects.

To support interpretation of the results, Figure 2 presents both the CRI of each attribute and estimated effects of individual attribute levels. The CRI analysis in Figure 2A showed that waiting time was the dominant driver of caregiver choices, accounting for approximately 60% of decision-making, far exceeding the relative contribution of telemedicine service type and reduction in hospital visits. Figure 2B further illustrates that variation in response time produced larger preference shifts than variation in service type or reduction in hospital visits.

Panel A shows the CRI of each telemedicine attribute. Panel B shows the estimated mean preference coefficients for each attribute level from the mixed multinomial logit model, with 95% confidence intervals; reference levels were fixed at zero. Abbreviations: AI, artificial intelligence; CRI, conditional relative importance.

**Figure 3.**
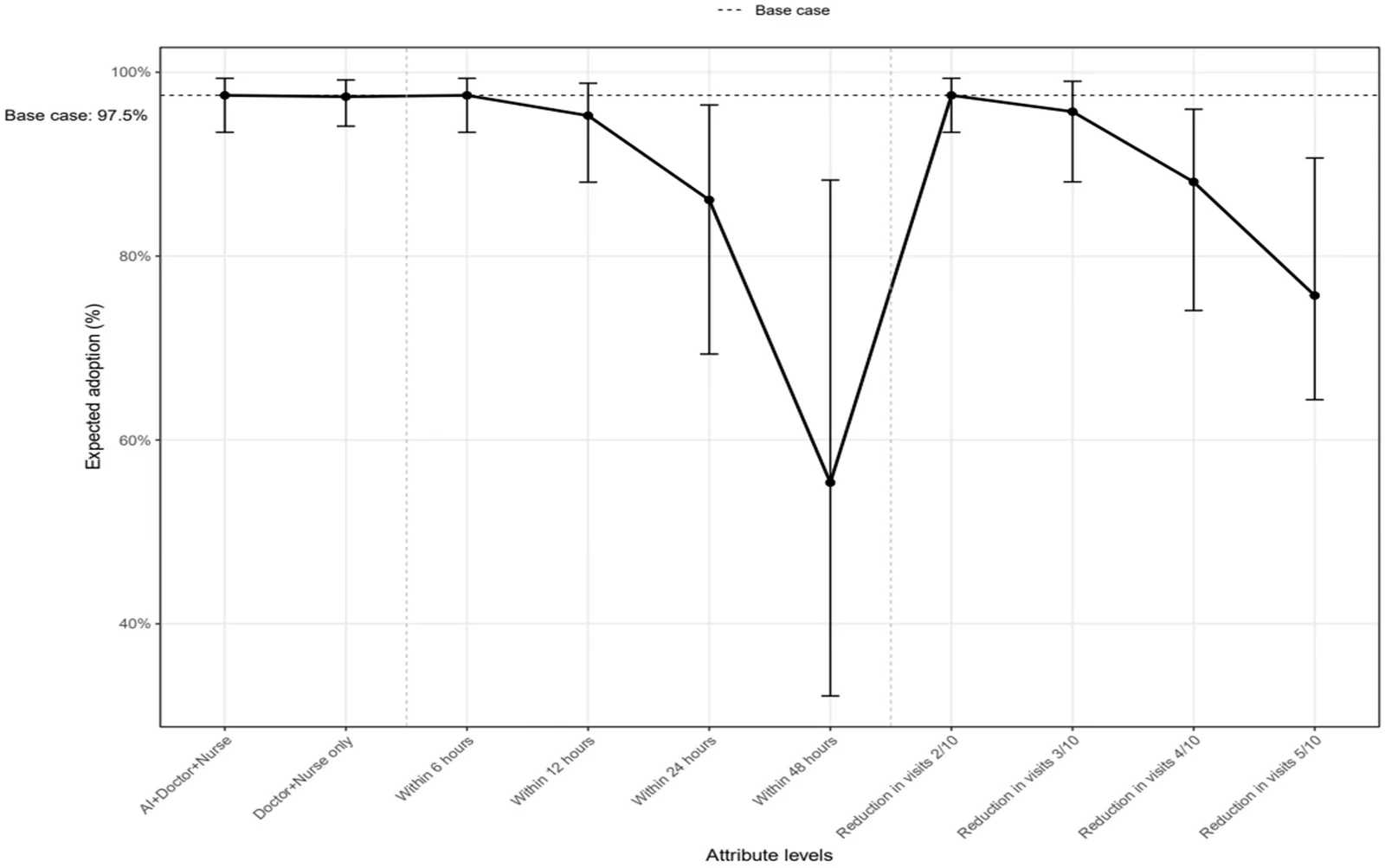
Predicted telemedicine uptake under one-way scenario variation using MMNL estimates.

The MMNL estimates were used to predict uptake under different telemedicine scenarios (Figure 3). In the base-case scenario, defined as AI-supported telemedicine with a response within 6 h and a 20% reduction in hospital visits, predicted uptake was 97.5% (95% simulated uncertainty interval, 93.1%-99.4%). Uptake was most sensitive to waiting time, decreasing to 95.3% for a 12-h response, 85.9% for a 24-h response, and 55.0% for a 48-h response. By contrast, predicted uptake changed little when the service was doctor- and nurse-supported rather than AI-supported (97.4%).

Points show the predicted uptake of the telemedicine service when one attribute level is varied at a time, while all other attributes are held at the base-case profile. Vertical lines indicate 95% uncertainty intervals, and the dashed horizontal line indicates the base-case predicted uptake. The base-case profile was defined as telemedicine with support from AI, doctor, and nurse, response within 6 h, and reduction in 2 of 10 hospital visits. Abbreviations: AI, artificial intelligence; MMNL, mixed multinomial logit.

Scenario-based comparisons suggested that respondents would accept a modest increase in waiting time for doctor- and nurse-supported rather than AI-supported telemedicine. The utility gain associated with doctor- and nurse-supported telemedicine (β = 0.92) exceeded the disutility of extending response time from 6 to 12 h (β = −0.62), but not the disutility of extending response time from 6 to 24 h (β = −1.37) (Supplemental File S5).

Although the MMNL suggested unobserved preference heterogeneity, additional subgroup or interaction analyses were not pursued because of the modest sample size.^36,37^ Internal validity and robustness were assessed through post hoc response-quality checks. One respondent showed no variation across tasks, and 2 were identified as speeders (completion times below the 5th percentile). Excluding these respondents did not materially affect preference estimates or conclusions (Supplemental File S6).

## Discussion

This study examined how PCGs of children receiving PPC at home valued characteristics of an asynchronous AI-supported telemedicine program. Caregivers expressed a strong stated preference for telemedicine across evaluated configurations, with high predicted uptake suggesting general acceptability in this home-based PPC context.

This finding aligns with the realities of caring for a child with LL or LT conditions at home. Families in PPC routinely manage complex symptoms, clinical uncertainty, and emotional distress outside regular clinical hours, creating a sustained need for timely communication and reassurance.^2^ Consistent with this context, response time emerged as the dominant attribute. Caregivers preferred services offering shorter response times, indicating that responsiveness may function as a prerequisite feature of telemedicine in PPC.

Although direct comparison is limited by the scarcity of DCEs in PPC, our findings are consistent with broader palliative and end-of-life preference literature. A recent systematic review identified only 17 palliative care DCEs, most conducted in adult settings rather than pediatric or telemedicine-specific contexts.^38^ Within this literature, service-delivery characteristics such as access, support, and organization of care appear to matter strongly to patients and caregivers.^38,39^ Similarly, telehealth studies suggest that digital models are most valued when they improve access, responsiveness, and continuity of support.^40^

Viewed from this perspective, telemedicine may be understood as a means of extending timely clinician presence into the home.^6,41^ By enabling rapid access to the care team and continuity of contact, telemedicine supports PCGs’ needs for communication and reassurance without weakening families’ perceived connection to clinicians.^1,8^ This relational dimension also helps explain preferences regarding service type. PCGs showed a modest but consistent preference for doctor- and nurse-supported telemedicine over AI-supported alternatives. This does not necessarily suggest rejection of AI, but rather indicates that caregivers value direct professional involvement, particularly when empathy, contextual judgment, and accountable decision-making are needed in situations of uncertainty or distress.^40,42^

A similar pattern was observed for hospital visits. Reductions in hospital attendance were not a consistent driver of caregiver choices, suggesting that PCGs did not prioritize efficiency or service substitution over opportunities for professional contact. In PPC, hospital visits and in-person encounters may represent not only clinical assessment but also reassurance and continuity through trusted relationships.^43^ The absence of a positive preference for reducing visits therefore suggests that telemedicine is valued primarily as a complement to existing care relationships rather than a replacement.^44^

Taken together, these findings indicate that PCG acceptance of telemedicine in PPC is shaped not only by operational efficiency but also by relational considerations. This has important implications for AI-supported telemedicine. Although AI is often promoted as a way to extend workforce capacity, address professional shortages, and reduce provider burden, these goals may conflict with caregiver preferences if AI is perceived as substituting for professional involvement.^45^

Addressing this tension may benefit from a considered approach to AI integration in PPC telemedicine. Framing AI primarily as clinician-facing infrastructure, for example, to support triage, prioritization, and information synthesis, may align more closely with caregiver expectations than presenting it as a substitute for professional contact.^46^ AI-supported workflows might also be oriented toward facilitating timely human responses rather than emphasizing automation.^47^

The integration of AI into PPC telemedicine may be further supported by codesign with PCGs and clinical teams, alongside transparent communication about the role and limits of AI.^48–50^ In a care context characterized by vulnerability and relational continuity, such approaches may help sustain trust and preserve the relationships families value.

## Strengths and Limitations

A strength of this study is that it addresses an important evidence gap by examining caregiver preferences for AI-supported telemedicine in PPC, where evidence has so far been largely limited to qualitative research. By using a DCE, this study moved beyond identifying themes alone and quantified the relative importance of service characteristics, the trade-offs caregivers were willing to make, and the predicted uptake of different telemedicine scenarios.

This study also has limitations. The sample size was modest, which restricted investigation of preference heterogeneity. Participants were recruited from a single tertiary center and had relatively high digital confidence, which may limit generalizability. Finally, because the study elicited stated preferences for a hypothetical telemedicine service, we could not assess real-world barriers such as internet connectivity, device access, or technology dropout, particularly among families living farther from the hospital.

## Conclusion

Families caring for children receiving PPC at home were open to AI-supported telemedicine, with acceptance driven by responsiveness and continuity with familiar clinicians rather than operational efficiency alone. These findings suggest that the role of AI in PPC telemedicine may be best understood as supporting, rather than substituting, clinician involvement. Aligning digital innovation with families’ priorities and relational needs may help ensure that AI-supported telemedicine strengthens, rather than compromises, family-centered care in PPC. Future research should include larger, more diverse populations across multiple care settings and regions, expand attribute sets, and explore subgroup-specific preferences to support equitable implementation.

## Supplemental Material

sj-docx-1-pal-10.1177_08258597261458091 - Supplemental material for Caregiver Preferences for AI-Supported Telemedicine in Pediatric Palliative Care: A Discrete Choice Experiment Supplemental material, sj-docx-1-pal-10.1177_08258597261458091 for Caregiver Preferences for AI-Supported Telemedicine in Pediatric Palliative Care: A Discrete Choice Experiment by Carlos Antonio Godoy Junior, Lucia Peñarrubia-San-Florencio, Silvia Ricart, Sergi Navarro Vilarrubí, Maria Rimblas Roure, Cristina Ruiz-Herguido, Arnau Valls-Esteve, Luis Pilli, Carin Uyl-de Groot, William Ken Redekop and Welmoed Kirsten van Deen in Journal of Palliative Care

sj-docx-2-pal-10.1177_08258597261458091 - Supplemental material for Caregiver Preferences for AI-Supported Telemedicine in Pediatric Palliative Care: A Discrete Choice Experiment Supplemental material, sj-docx-2-pal-10.1177_08258597261458091 for Caregiver Preferences for AI-Supported Telemedicine in Pediatric Palliative Care: A Discrete Choice Experiment by Carlos Antonio Godoy Junior, Lucia Peñarrubia-San-Florencio, Silvia Ricart, Sergi Navarro Vilarrubí, Maria Rimblas Roure, Cristina Ruiz-Herguido, Arnau Valls-Esteve, Luis Pilli, Carin Uyl-de Groot, William Ken Redekop and Welmoed Kirsten van Deen in Journal of Palliative Care

sj-docx-3-pal-10.1177_08258597261458091 - Supplemental material for Caregiver Preferences for AI-Supported Telemedicine in Pediatric Palliative Care: A Discrete Choice Experiment Supplemental material, sj-docx-3-pal-10.1177_08258597261458091 for Caregiver Preferences for AI-Supported Telemedicine in Pediatric Palliative Care: A Discrete Choice Experiment by Carlos Antonio Godoy Junior, Lucia Peñarrubia-San-Florencio, Silvia Ricart, Sergi Navarro Vilarrubí, Maria Rimblas Roure, Cristina Ruiz-Herguido, Arnau Valls-Esteve, Luis Pilli, Carin Uyl-de Groot, William Ken Redekop and Welmoed Kirsten van Deen in Journal of Palliative Care

sj-docx-4-pal-10.1177_08258597261458091 - Supplemental material for Caregiver Preferences for AI-Supported Telemedicine in Pediatric Palliative Care: A Discrete Choice Experiment Supplemental material, sj-docx-4-pal-10.1177_08258597261458091 for Caregiver Preferences for AI-Supported Telemedicine in Pediatric Palliative Care: A Discrete Choice Experiment by Carlos Antonio Godoy Junior, Lucia Peñarrubia-San-Florencio, Silvia Ricart, Sergi Navarro Vilarrubí, Maria Rimblas Roure, Cristina Ruiz-Herguido, Arnau Valls-Esteve, Luis Pilli, Carin Uyl-de Groot, William Ken Redekop and Welmoed Kirsten van Deen in Journal of Palliative Care

sj-docx-5-pal-10.1177_08258597261458091 - Supplemental material for Caregiver Preferences for AI-Supported Telemedicine in Pediatric Palliative Care: A Discrete Choice Experiment Supplemental material, sj-docx-5-pal-10.1177_08258597261458091 for Caregiver Preferences for AI-Supported Telemedicine in Pediatric Palliative Care: A Discrete Choice Experiment by Carlos Antonio Godoy Junior, Lucia Peñarrubia-San-Florencio, Silvia Ricart, Sergi Navarro Vilarrubí, Maria Rimblas Roure, Cristina Ruiz-Herguido, Arnau Valls-Esteve, Luis Pilli, Carin Uyl-de Groot, William Ken Redekop and Welmoed Kirsten van Deen in Journal of Palliative Care

sj-docx-6-pal-10.1177_08258597261458091 - Supplemental material for Caregiver Preferences for AI-Supported Telemedicine in Pediatric Palliative Care: A Discrete Choice Experiment Supplemental material, sj-docx-6-pal-10.1177_08258597261458091 for Caregiver Preferences for AI-Supported Telemedicine in Pediatric Palliative Care: A Discrete Choice Experiment by Carlos Antonio Godoy Junior, Lucia Peñarrubia-San-Florencio, Silvia Ricart, Sergi Navarro Vilarrubí, Maria Rimblas Roure, Cristina Ruiz-Herguido, Arnau Valls-Esteve, Luis Pilli, Carin Uyl-de Groot, William Ken Redekop and Welmoed Kirsten van Deen in Journal of Palliative Care
