# Supplementary material for: Caregiver Preferences for AI-Supported Telemedicine in Pediatric Palliative Care: A Discrete Choice Experiment
Source: J Palliat Care. 2026 Jun 10;41(4):473–83. doi: 10.1177/08258597261458091 (PMC13550745; doi:10.1177/08258597261458091)
Supplement: Supplementary file 1 [file sj-docx-1-pal-10.1177_08258597261458091.docx]

# Supplementary File 1

# Reporting guidelines: completed DIRECT checklist for reporting of this discrete choice experiment

Assessment of compliance with the Direct checklist (Ride et al., 2024) for the present discrete choice experiment.

| **Item** | **Page and paragraph** | **Reported** |
| --- | --- | --- |
| **Purpose and rationale** |  |  |
| 1. Describe the real-world context and decision-maker that the hypothetical choice context seeks to replicate or inform | Introduction, paras 1–5; Methods, “Study design and setting” | Yes |
| 2. Provide a rationale for using a DCE to answer the research question | Methods, “Study design and setting,” para 1 | Yes |
| **Attributes and levels** |  |  |
| 3. Describe how attributes and levels were derived (e.g. literature review, interviews, focus groups, expert input) | Methods, “DCE methodology,” paras 1–2; Supplementary File 2 | Yes |
| 4. Provide the final list of attributes and levels | Table 1; Methods, “DCE methodology,” para 2 | Yes |
| **Experimental design** |  |  |
| 5. Report the number of alternatives per choice set and whether they were labelled or unlabelled | Methods, “DCE methodology,” final para; Figure 1 | Yes |
| 6. Describe response options (e.g. forced choice, opt-out, status quo) | Methods, “DCE methodology,” final para; Figure 1 | Yes |
| 7. Describe the type of experimental design (e.g. orthogonal, D-efficient, Bayesian efficient, partial profile) | Methods, “DCE methodology,” para beginning “The number of attributes and levels…” | Yes |
| 8. Describe which effects are identified in the design (e.g. main effects, higher order interactions, functional form) | Methods, “DCE methodology,” same paragraph | Yes |
| 9. Describe the number of choice sets, blocks and choice sets per block | Methods, “DCE methodology,” final para | Yes |
| 10. Indicate how the experimental design was obtained (software, catalogue, other) | Methods, “DCE methodology,” paragraph describing Ngene | Yes |
| **Survey design** |  |  |
| 11. Provide a sample choice set and the instructions and background information given to respondents (e.g. providing the survey as an appendix) | Figure 1; Methods, “Survey instrument”; Supplementary File 4 | Yes |
| 12. Report any randomisation (e.g. choice set order, attribute order, alternative order, framing effects) | Methods, “DCE methodology,” final para | Yes |
| 13. Describe what was checked in piloting (e.g. understanding, respondent burden, timing, wording) | Methods, “Survey instrument,” final paragraph | Yes |
| 14. Report whether information from the pilot was used to update the experimental design (e.g. priors, functional form of attributes) or survey design | Methods, “Survey instrument,” final paragraph | Yes |
| **Sample and data collection** |  |  |
| 15. Report respondent inclusion/exclusion criteria | Methods, “Participants and recruitment,” para 1 | Yes |
| 16. Describe how data were collected (e.g. mail, personal interview, web survey) | Methods, “Participants and recruitment,” para 1; “Survey instrument” | Yes |
| 17. Report the response rate or cooperation rate, if possible | Results, para 1 | Yes |
| 18. Report the final sample size and how the sample size was determined | Methods, “Participants and recruitment,” para 1; Results, para 1 | Yes |
| 19. Describe respondent characteristics and representativeness of target population, if known | Results, para 2; Table 2; Discussion, “Strengths and limitations” | Yes |
| **Econometric analysis** |  |  |
| 20. Indicate coding of data (e.g. effects, dummy, continuous) including definitions | Methods, “Statistical analysis,” para 1 | Yes |
| 21. Report whether any respondents were removed and why (e.g. suspected fraudulent responses, rationality tests) | Methods, “Conditional relative importance and uptake prediction”; Results, final paragraph | Yes |
| 22. Provide the rationale for model choice (e.g. conditional logit, mixed logit, latent class) and assumptions (e.g. error variance) | Methods, “Statistical analysis,” paras 1–2 | Yes |
| 23. Report model specification | Methods, “Statistical analysis,” paras 1–2 | Yes |
| **Reporting of results** |  |  |
| 24. Report the model performance, goodness of fit (if comparing models) | Results, para before Table 3; Table 3 | Yes |
| 25. Describe methods used for analysis of model results (e.g. calculation of marginal rate of substitution, attribute relative importance, welfare gain) | Methods, “Conditional relative importance and uptake prediction” | Yes |
| 26. Report measures of precision for the output(s) of interest (e.g. confidence intervals) and how these were derived | Methods, “Statistical analysis”; Table 3; Results text | Yes |
