# Supplementary material for: Caregiver Preferences for AI-Supported Telemedicine in Pediatric Palliative Care: A Discrete Choice Experiment
Source: J Palliat Care. 2026 Jun 10;41(4):473–83. doi: 10.1177/08258597261458091 (PMC13550745; doi:10.1177/08258597261458091)
Supplement: Supplementary file 2 [file sj-docx-2-pal-10.1177_08258597261458091.docx]

# **Supplementary File 2**

# **Candidate attributes considered during DCE development and rationale for final attribute selection**

| **Candidate attribute** | **Examples of levels/concepts considered during development** | **Retained in final DCE** | **Rationale for inclusion or exclusion** |
| --- | --- | --- | --- |
| **Type of telemedicine service** | AI-supported telemedicine with clinician oversight; telemedicine supported by doctor and nurse only | **Yes** | Retained because it directly captured the central study question: whether caregivers distinguish between AI-supported and clinician-led telemedicine. It was highly relevant to the proof-of-concept service under investigation, conceptually distinct from the other attributes, and important for informing service design. |
| **Waiting time for healthcare provider response** | Within 6, 12, 24, or 48 hours | **Yes** | Retained because timely access to professional advice and reassurance emerged as a key theme from the literature and qualitative work. It was expected to be salient to caregivers, actionable for service planning, and suitable for expressing meaningful trade-offs within the DCE. |
| **Reduction in hospital visits** | Avoid 2, 3, 4, or 5 of 10 visits | **Yes** | Retained because reducing unnecessary hospital attendance was considered a plausible potential benefit of asynchronous telemedicine. It represented a service consequence that families could trade off against professional involvement and response time. |
| Mode of communication | Written messaging only; messaging plus telephone/video follow-up | No | Excluded because the study focused specifically on an asynchronous telemedicine concept. Including communication mode would have broadened the intervention beyond the intended service model and risked conflating asynchronous care with synchronous telehealth features. |
| Scope of AI support | AI used only for triage; AI provides automated symptom guidance; AI more active in decision support | No | Excluded because more granular distinctions in AI functionality were considered difficult to communicate clearly in a short survey and may have imposed a high cognitive burden on respondents. The final design therefore simplified this dimension into a single AI-supported versus clinician-only service contrast. |
| Level of clinician oversight | Full clinician review of all cases; clinician review only for selected escalations | No | Excluded because this concept was closely tied to the AI-service description and might have confused respondents if treated as a separate attribute. Instead, clinician oversight was standardized in the background explanation for the AI-supported alternative. |
| Emotional support or reassurance | More reassurance; less reassurance; formal psychosocial support features | No | Excluded because this concept was important but difficult to operationalize as a discrete, measurable service attribute with realistic levels. It was also considered likely to be interpreted inconsistently across respondents. |
| Data privacy and confidentiality | Standard data protection; enhanced privacy safeguards | No | Excluded because, although relevant to digital health, it was not the primary decision focus of the proof-of-concept service and may have functioned as a dominant attribute, reducing respondents’ ability to trade across other service characteristics. |
| Out-of-pocket cost | Monthly fee; no fee; co-payment levels | No | Excluded because the study was conducted in a publicly oriented care context where introducing hypothetical personal payment could have reduced realism. Without a cost attribute, willingness-to-pay analyses were not possible, but the design remained more closely aligned with the real service context. |
| Ease of platform use / technical usability | Easy to use; moderate effort; difficult to use | No | Excluded because usability was considered an implementation issue better explored in real-world testing rather than a defining service attribute at the proof-of-concept preference stage. In addition, respondents had not used the platform in practice. |
| Availability outside working hours | Service available during office hours only; evenings/weekends included | No | Excluded because this concept partially overlapped with response time and might have introduced ambiguity about whether caregivers were valuing speed, timing, or workforce availability. Response time was judged the clearer and more policy-relevant measure. |
| Type of health professional responding | Doctor only; nurse only; doctor and nurse team | No | Excluded because separating professional role from AI support would have substantially expanded the design. The final telemedicine-service attribute captured a simpler and more interpretable contrast between clinician-led and AI-supported care models. |
