# Supplementary material for: Caregiver Preferences for AI-Supported Telemedicine in Pediatric Palliative Care: A Discrete Choice Experiment
Source: J Palliat Care. 2026 Jun 10;41(4):473–83. doi: 10.1177/08258597261458091 (PMC13550745; doi:10.1177/08258597261458091)
Supplement: Supplementary file 3 [file sj-docx-3-pal-10.1177_08258597261458091.docx]

# **Supplementary File 3**

# **Ngene D-efficient Designs for Alternating Choice Tasks A and B**

**A**

design

;alts=altA*, altB*, altC*

;eff=(mnl,d, fixed)

;alg=mfederov

;rows=12

;block=2

;model:

U(altA) =	b1.dummy[0]	*type[2,1]

+ b2.dummy[-0.2|-0.15|-0.1] *waittime [4,3,2,1]

+ b3.dummy[0.2|0.1|0.05]	*efficacy [4,3,2,1]

/

U(altB) =	b1.dummy[0]	*type[2,1]

+ b2.dummy[-0.2|-0.15|-0.1] *waittime [4,3,2,1]

+ b3.dummy[0.2|0.1|0.05]	*efficacy [4,3,2,1]

/

U(altC) =	b1.dummy[0]	*type[2,1]

+ b2.dummy[-0.2|-0.15|-0.1] *waittime [4,3,2,1]

+ b3.dummy[0.2|0.1|0.05]	*efficacy [4,3,2,1]

$

**B**

design

;alts=altA*, altB*, altC

;eff=(mnl,d, fixed)

;alg=mfederov

;rows=12

;block=2

;model:

U(altA) =	b1.dummy[0]	*type[2,1]

+ b2.dummy[-0.2|-0.15|-0.1] *waittime [4,3,2,1]

+ b3.dummy[0.2|0.1|0.05]	*efficacy [4,3,2,1]

/

U(altB) =	b1.dummy[0]	*type[2,1]

+ b2.dummy[-0.2|-0.15|-0.1] *waittime [4,3,2,1]

+ b3.dummy[0.2|0.1|0.05]	*efficacy [4,3,2,1]

/

U(altC) =	b0

$
