# Supplementary material for: Caregiver Preferences for AI-Supported Telemedicine in Pediatric Palliative Care: A Discrete Choice Experiment
Source: J Palliat Care. 2026 Jun 10;41(4):473–83. doi: 10.1177/08258597261458091 (PMC13550745; doi:10.1177/08258597261458091)
Supplement: Supplementary file 5 [file sj-docx-5-pal-10.1177_08258597261458091.docx]

# **Supplementary File 5**

# **Scenario-based trade-off comparisons derived from the mixed multinomial logit model (MMNL)**

| Scenario comparison | Utility estimate (β) | 95% CI | Interpretation |
| --- | --- | --- | --- |
| Doctor- and nurse-led telemedicine rather than AI-supported telemedicine | 0.92 | 0.18 to 1.65 | Respondents showed a modest preference for clinician-led telemedicine over AI-supported telemedicine |
| Waiting time extended from 6 to 12 hours | -0.62 | -1.25 to 0.02 | The disutility associated with waiting 12 rather than 6 hours was smaller in magnitude than the utility gain for clinician-led telemedicine |
| Waiting time extended from 6 to 24 hours | -1.37 | -2.28 to -0.46 | The disutility associated with waiting 24 rather than 6 hours was greater in magnitude than the utility gain for clinician-led telemedicine |
| Waiting time extended from 6 to 48 hours | -3.55 | -5.81 to -1.30 | A delay to 48 hours was associated with a large decrease in utility, indicating poor acceptability relative to rapid response |

# **Trade-off interpretation**

| Trade-off scenario | Net utility comparison | Interpretation |
| --- | --- | --- |
| Clinician-led telemedicine with response within 12 hours versus AI-supported telemedicine with response within 6 hours | 0.92 - 0.62 = **+0.30** | On average, respondents would accept a modest increase in waiting time from 6 to 12 hours in exchange for a doctor- and nurse-led service |
| Clinician-led telemedicine with response within 24 hours versus AI-supported telemedicine with response within 6 hours | 0.92 - 1.37 = **-0.45** | On average, respondents would not accept a longer increase in waiting time from 6 to 24 hours in exchange for a doctor- and nurse-led service |
| Clinician-led telemedicine with response within 48 hours versus AI-supported telemedicine with response within 6 hours | 0.92 - 3.55 = **-2.63** | A delay to 48 hours outweighed the preference for clinician-led telemedicine by a large margin |

**Note:** These comparisons are based on MMNL coefficient differences and are intended to support interpretation of relative trade-offs across selected service features. They do not represent willingness-to-pay estimates or formal marginal rates of substitution.
