# Supplementary material for: Caregiver Preferences for AI-Supported Telemedicine in Pediatric Palliative Care: A Discrete Choice Experiment
Source: J Palliat Care. 2026 Jun 10;41(4):473–83. doi: 10.1177/08258597261458091 (PMC13550745; doi:10.1177/08258597261458091)
Supplement: Supplementary file 6 [file sj-docx-6-pal-10.1177_08258597261458091.docx]

# **Supplementary File 6**

# **Internal validity**

Internal validity and robustness were assessed through post-hoc response-quality checks, including completion time and within-respondent choice variation. One respondent showed no variation across tasks, and two were identified as speeders (completion times below the 5th percentile). Excluding these respondents did not materially affect preference estimates or conclusions.

**Exclude only no-variation responder**

| Attribute | Level | β Coefficient | SE | p-value |
| --- | --- | --- | --- | --- |
| Type of Telemedicine Service | AI + Doctor + Nurse (ref.)  Doctor and Nurse only | ref.  0.48 | —  0.21 | —  0.02 |
|  |  |  |  |  |
| Response Time | Within 6 hours (ref.)  Within 12 hours | ref.  -0.70 | —  0.19 | —  <0.01 |
|  | Within 24 hours | -0.98 | 0.35 | <0.01 |
|  | Within 48 hours | -1.62 | 0.48 | <0.001 |
|  |  |  |  |  |
| Reduction in Hospital Visits | Avoids 2 of 10 visits (ref.)  Avoids 3 of 10 visits | ref.  -0.56 | —  0.31 | —  0.10 |
|  | Avoids 4 of 10 visits | -0.84 | 0.41 | 0.06 |
|  | Avoids 5 of 10 visits | -0.69 | 0.45 | 0.17 |
|  |  |  |  |  |
| Opt-out |  | -3.17 | 0.49 | <0.001 |

**Exclude speeders**

| Attribute | Level | β Coefficient | SE | p-value |
| --- | --- | --- | --- | --- |
| Type of Telemedicine Service | AI + Doctor + Nurse (ref.)  Doctor and Nurse only | ref.  0.46 | —  0.20 | —  0.02 |
| Response Time | Within 6 hours (ref.)  Within 12 hours | ref.  -0.62 | —  0.19 | —  <0.01 |
|  | Within 24 hours | -0.95 | 0.34 | <0.01 |
|  | Within 48 hours | -1.53 | 0.46 | <0.001 |
|  |  |  |  |  |
| Reduction in Hospital Visits | Avoids 2 of 10 visits (ref.)  Avoids 3 of 10 visits | ref.  -0.53 | —  0.32 | —  0.10 |
|  | Avoids 4 of 10 visits | -0.82 | 0.42 | 0.05 |
|  | Avoids 5 of 10 visits | -0.66 | 0.47 | 0.16 |
|  |  |  |  |  |
| Opt-out |  | -3.16 | 0.48 | <0.001 |

**Exclude both (combined)**

| Attribute | Level | β Coefficient | SE | p-value |
| --- | --- | --- | --- | --- |
| Type of Telemedicine Service | AI + Doctor + Nurse (ref.)  Doctor and Nurse only | ref.  0.48 | —  0.21 | —  0.02 |
| Response Time | Within 6 hours (ref.)  Within 12 hours | ref.  -0.70 | —  0.19 | —  <0.001 |
|  | Within 24 hours | -0.98 | 0.35 | <0.01 |
|  | Within 48 hours | -1.62 | 0.48 | <0.001 |
|  |  |  |  |  |
| Reduction in Hospital Visits | Avoids 2 of 10 visits (ref.)  Avoids 3 of 10 visits | ref.  -0.56 | —  0.34 | —  0.10 |
|  | Avoids 4 of 10 visits | -0.84 | 0.45 | 0.06 |
|  | Avoids 5 of 10 visits | -0.69 | 0.50 | 0.16 |
|  |  |  |  |  |
| Opt-out |  | -3.17 | 0.49 | <0.001 |
