# Supplementary material for: Caregiver Preferences for AI-Supported Telemedicine in Pediatric Palliative Care: A Discrete Choice Experiment
Source: J Palliat Care. 2026 Jun 10;41(4):473–83. doi: 10.1177/08258597261458091 (PMC13550745; doi:10.1177/08258597261458091)
Supplement: Supplementary file 7 [file sj-docx-4-pal-10.1177_08258597261458091.docx]

# **Supplementary File 4**

# **English version of the full DCE survey (29 pages)**
